# Effectiveness and safety of traditional Chinese medicines for pulmonary heart disease: A protocol for systematic review and meta-analysis

**DOI:** 10.1097/MD.0000000000024131

**Published:** 2021-01-08

**Authors:** Xinyu Hu, Lulu Li, Yuanying Song, Yun Lu

**Affiliations:** a Chengdu University of Traditional Chinese Medicine, School of Clinical Medicine; b Hospital of Chengdu University of Traditional Chinese Medicine, Chengdu, Sichuan, China.

**Keywords:** GRADE, meta-analysis, pulmonary heart disease, systematic review, traditional Chinese medicines

## Abstract

**Background:**

Previous review indicate that the effect of traditional Chinese medicines (TCM) on pulmonary heart disease (PHD) remains uncertainty. Therefore, we designed this study to systematically evaluate the effectiveness and safety of TCM in the treatment of PHD.

**Methods:**

Nine online databases will be searched from inception to October 01, 2021, and we will not restrict the language on included trials. Randomized controlled trials that included patients with PHD receiving TCM therapy vs a control group will be included. Two of us will perform independently the selection of studies, risk of bias assessment, and data extraction. The RevMan V.5.2 software with fixed effects model or random effects model will be used to syntheses the data, according to the heterogeneity test to conduct the data synthesis. The dichotomous data and the continuous data will be presented with risk ratios with 95% confidence intervals and weighted mean differences or standardized mean differences with 95% confidence intervals. And we will use the Grading of Recommendations Assessment, Development, and Evaluation system to evaluate the evidence quality.

**Result:**

This study will assess effects and safety for TCM on PHD.

**Conclusion:**

The conclusion of this study will provide evidence to prove the safety and effectiveness of TCM on PHD.

**Trial registration number:**

INPLASY2020120024.

## 1 Introductions

Pulmonary heart disease (PHD) is a type of heart disease that causes abnormal lung function and tissue structure lesions caused by lung tissue, bronchus, pulmonary vascular disease, and respiratory regulation disorders, thereby causing heart damage.^[1,2]^ Slow, can be divided into acute and chronic cor pulmonale. Chronic pulmonary heart disease is mainly caused by chronic diseases such as lung tissue, bronchi, and thorax that cause increased pulmonary circulatory resistance and pulmonary hypertension, which leads to enlargement of the right heart or accompanied by cardiac insufficiency. Acute pulmonary heart disease is mainly due to embolism in the main trunk or main branches of the pulmonary artery, and even the pulmonary artery. The pressure suddenly increases, causing acute dilation of the right heart and acute heart failure.^[3,4]^

Traditional Chinese medicine is the heritage and essence of China for 5000 years and has improved the health of the Chinese people. Studies have found that traditional Chinese medicine has a certain effect on the stable phase of PHD,^[5–12]^ but the results of different studies are inconsistent. And the drugs used in different institutes are different. Therefore, we designed this systematic review and meta-analysis to evaluate whether traditional Chinese medicine is safe and effective for the stable phase of PHD from a macro perspective.

## 2 Methods

We completed the protocol of systematic review and meta-analysis according to the PRISMA-P guidelines.^[13]^

### 2.1 Criteria for inclusion

These standards are predesignated according to the PICOS standard, which involves patients or populations, interventions, comparisons, results, and study design.

### 2.2 Types of participants

The trial included patients of any age, regardless of gender and stage of cor pulmonale. All participants must be diagnosed from the domestic literature based on the 1977/1980 Chinese pulmonary heart disease diagnostic criteria and the English literature based on the WHO pulmonary heart disease diagnostic criteria.

### 2.3 Types of interventions

We will include all trials using traditional Chinese medicine (experimental group) and non-Chinese medicine (control group) to treat pulmonary heart disease, without limiting the types and applicable methods of traditional Chinese medicine. Trials initiated by pharmaceutical companies or clinicians are also included.

### 2.4 Types of comparator(s)/control

Control groups with placebo or other active treatments are also included. Active treatment includes drugs such as antibiotics, analgesics, and corticosteroids.

### 2.5 Types of outcome indicators

#### 2.5.1 Primary outcomes

(1) Effective rate: refers to the ratio of responders to the total patients, based on the efficacy standards of the Chinese National Conference on Cor Pulmonale in 1977/1980
(2) Death

#### 2.5.2 Secondary outcomes

Quality of life score, Partial Pressure of Oxygen and Carbon Dioxide, Adverse Events.

### 2.6 Types of studies

We will include randomized controlled trial. Multiweapon tests that meet the above criteria will be included. For crossover experiments, data will only be extracted from the first stage to avoid potential carry-over effects. Another study design will be excluded.

### 2.7 Search methods for identification of studies

#### 2.7.1 Electronic searches

We will search 9 databases from the inception dates to October 01, 2021: WanFang, China Science and Technology Journal Database (VIP), China National Knowledge Infrastructure (CNKI), PubMed, Embase, the Cochrane Library, and China Biomedical Literature (CBM) databases. The searching strategy of PubMed is presented in Table 1.

**Table 1:** Search strategy used in Pubmed database.

Number	Search terms
1	Randomized controlled trial [All Fields]
2	Controlled clinical trial [All Fields]
3	Randomized [All Fields]
4	Randomised [All Fields]
5	Placebo [All Fields]
6	Randomly [All Fields]
7	Trial [All Fields]
8	Groups [All Fields]
9	or/1–8
10	Herb [Title/Abstract]
11	Herbal [Title/Abstract]
12	Chinese medicine [Title/Abstract]
13	Combinations of TCMs [Title/Abstract]
14	Plant [Title/Abstract]
15	Or/10–14
16	PHD [Title/Abstract]
17	Chronic pulmonary heart disease [Title/Abstract]
18	Chronic cor pulmonale [Title/Abstract]
19	Pulmonary hypertension [Title/Abstract]
20	Chronic obstructive pulmonary disease [Title/Abstract]
21	COPD [Title/Abstract]
22	Or/15–21
23	9 and 15 and 22

#### 2.7.2 Searching other resources

We incorporate searches into the research and reference lists of relevant systematic reviews or meta-analysis to ensure a comprehensive search. At the same time, we will also search the WHO Clinical Trial Registry and the Chinese Clinical Trial Registry to ensure that relevant grey literature is included.

### 2.8 Data collection and analysis

#### 2.8.1 Selection of studies

**Figure 1:**
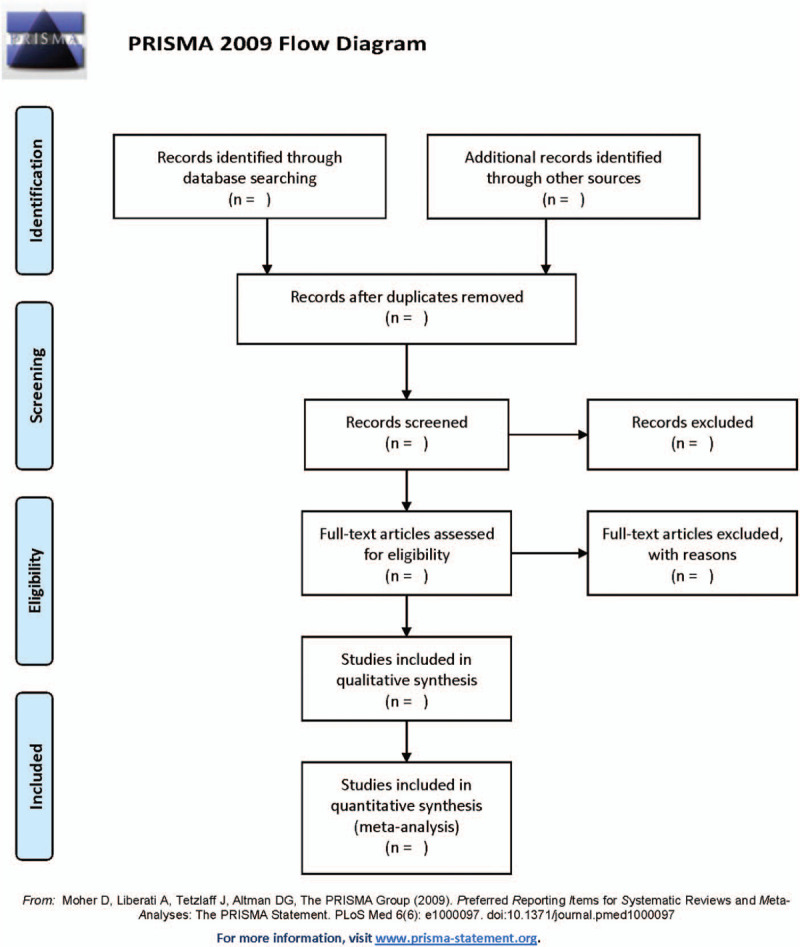
Flow diagram of study selection process.

We will use Endnote software (X8 version) to manage electronic search research and data obtained from other sources. First, we import the search results into the software, and then eliminate duplicate documents, which are mainly based on the author, title, and abstract (the same content in different languages or different publishing formats, or 2 articles written in the same experiment from different aspects). Secondly, we will eliminate the obviously nonconforming studies based on the title and abstract. This step is carried out independently by 2 researchers based on the inclusion and exclusion criteria. Finally, we will read the full text of all remaining studies to determine if it is the research we need. The entire process is completed by 2 different researchers. If there is disagreement, we will resolve it through discussion. When the opinions are still inconsistent, the third person will decide. The research selection process and results will be presented in the form of a flowchart (Fig. 1).

#### 2.8.2 Data extraction and management

Before data extraction, we will use Excel to establish a standard data extraction table, which includes the following information: basic information (including journal, first author, publication time, etc), research characteristics (research design, grouping, whether it is multicenter, random methods, blinding, analysis methods, research purposes, etc), research quality evaluation (evaluation of research quality, bias risk assessment, etc), participants (age, gender, race, country, diagnosis, disease course, etc), intervention and control (intervention methods), drug name, medication method, drug dosage, medication time, duration of a course of treatment, name and type of control, additional treatment, etc), outcome measurement (primary and secondary results, evaluation time, follow-up time, etc), outcome (average, SD, intervention, follow-up, total sample size, etc). The data extraction work was completed by 2 researchers simultaneously and independently, and cross-checked each other after completion. If there is a difference, it will be resolved through discussion among all researchers. The third inspector will check the entered data to ensure data consistency and correct data entry errors.

#### 2.8.3 Assessment of risk of bias in included studies

The risk of bias for included studies will be evaluated using the Cochrane Collaboration's tool for assessing risk of bias.^[14]^ For each domain, we will categories the risk of bias as low, unclear, or high risk of bias. The domains for risk of bias are as follows:

1. Selection bias (randomization sequence generation and allocation concealment);
2. Performance bias (blinding of participants and personnel);
3. Detection bias (blinding of outcome assessment);
4. Attrition bias (incomplete outcome data).
5. Reporting bias (selective reporting);
6. Other bias (including baseline imbalance, claimed to have been fraudulent, differential diagnostic activity, and contamination).

#### 2.8.4 Dealing with missing data

We will contact the author to obtain the original data. If the author cannot be contacted or the data is missing, this study will be excluded and the remaining studies will be synthesized.

#### 2.8.5 Assessment of heterogeneity

We will use chi-square (*X*^2^) to assess statistical heterogeneity, according to the Cochrane Handbook,^[15]^ if a *P* value of less than .10 is considered significant. In addition, the *I*^2^ value of RevMan V.5.2 will be used to quantify the impact of statistical heterogeneity on meta-analysis. The interpretation of *I*^2^ is roughly as follows: 0% to 40%: may not be important; 30% to 60%: may represent moderate heterogeneity; 50% to 90%: may show significant heterogeneity; 75% to 100%: Considerable heterogeneity.^[15]^ In addition, the importance of the observed value of *I*^2^ depends on 2 aspects:

(1) the strength of the evidence of heterogeneity;
(2) the magnitude and direction of the impact [such as the *P*-value of the chi-square test, or the confidence interval (CI) of I^2^].^[15]^

### 2.9 Measures of treatment effect

#### 2.9.1 Data synthesis

Before integrating the data, we will unify the unit of each result of different experiments according to the international unit system. All data will be synthesized using RevMan5.2 or STATA software. The 95% CIs of the risk ratio/odds ratio will give the results of the dichotomous data analysis, while the continuous results will use the 95% CI of the mean difference/standardized mean difference investigate. When *I*^2^ < 75% comes from the heterogeneity test, the data will be synthesized and analyzed. When the heterogeneity test shows slight or no statistical heterogeneity in these trials (*I*^2^ value is not less than 40%), we will use a fixed-effects model for the combined data. When significant heterogeneity is detected (*I*^2^ 40%, <75%), a random effects model will be used for data synthesis. If there is considerable heterogeneity in the trial, no meta-analysis is performed. In this case, we will try to determine the source of heterogeneity from both clinical and methodological aspects, and provide a qualitative summary. When more than 10 trials are included, a funnel chart will be generated to observe the reported deviation.

#### 2.9.2 Subgroup analysis and meta-regression

If enough trials are included, we will use STATA software to explore the following possible sources of heterogeneity by performing subgroup analysis or meta-regression on changes in trial participant characteristics, traditional Chinese medicine treatment, sample size, methodology, missing data, etc.

#### 2.9.3 Sensitivity analysis

Sensitivity analysis will be used to check the stability of major decisions made during the review process. Several decision nodes will be considered in the system review process, such as small sample size, lack of method, and lack of data. The results of the sensitivity analysis will be presented in the form of a summary table. As the sensitivity analysis results show, the risk of bias in the review process will be discussed.

#### 2.9.4 Evidence quality evaluation

We will use the Grading of Recommendations Assessment, Development, and Evaluation (GRADE) system to assess the quality of evidence for each outcome.^[16]^ According to the GRADE rating standards, the evidence quality will be rated with “high,” “moderate,” “low” or “very low.” The evaluation of evidence quality is mainly based on the following 5 aspects: the risk of bias of included studies, inconsistency of different research, indirectness of evidence, imprecision of results, publication bias of randomized controlled trials, large effect of sample, dose response of traditional Chinese medicines, and all plausible confounding.^[16,17]^ The results of GRADE system evaluation will be summarized with a table to be presented in the final report.^[17]^

## Author contributions

**Funding acquisition:** Yun Lu.

**Methodology:** Lulu Li.

**Writing – original draft:** Xinyu Hu.

**Writing – review & editing:** Yuanying Song, Yun Lu.
